# Risk Factors Associated with the Occurrence of the Second-Litter Syndrome in Sows in Southeastern Mexico

**DOI:** 10.1155/2013/969620

**Published:** 2013-10-29

**Authors:** José C. Segura Correa, Alejandro Alzina-López, Ronald H. Santos-Ricalde

**Affiliations:** Facultad de Medicina Veterinaria y Zootecnia, Universidad Autónoma de Yucatán, Km 15.5 Carretera Mérida-Xmatkuil, Apdo. Postal 4-116, Mérida, YUC, Mexico

## Abstract

The objective was to estimate the incidence of and to determine the effect of some risk factors on the decrease of litter size at parity 2 of sows in three commercial farms in Yucatan, Mexico. Data on 8,592 farrowing records of 4,296 sows were analyzed using a binomial logistic regression procedure. The model included the fixed effect of farm (1, 2, and 3), year of farrowing (2003–2011), season of farrowing (dry, rainy, and windy), number of pigs born alive at first parity (<9, 9-10, 11-12, and >12 piglets), lactation length (<18, 18–24, and >24 days), and weaning to conception intervals (<4, 4–11, and >11 days). Fifty-five point eight percent of all sows presented a reduced or similar litter size at parity 2. The odds of decrease in the second litter size were 1.56 and 2.01 for farms 2 and 3, respectively. Higher odds were found for sows farrowing during the rainy and dry seasons (1.20 and 1.24, resp.) and for sows with large litters at parity 1 (>12 piglets, odds = 33.2). Sows with weaning to conception intervals <4 days and between 4 and 11 days had higher odds of a decrease in the second litter (1.78 and 2.74 pigs, resp.).

## 1. Introduction

Litter size commonly increases with the parity number in sows [1]. However, in some sows the number of piglets in the second litter is lower or similar to that of the first litter, phenomenon known as second-litter syndrome [2, 3]. The second-litter syndrome negatively affects the pregnancy rate of second parity sows and sows productive lifetime in the farm, since reproductive failure is one of the main reasons for culling young sows [4, 5]. Therefore, the estimation of and the identification of risk factors associated with the second litter-syndrome may be of great help in planning better management strategies to improve the second-parity reproductive performance. Litter size at first parity is one of the main risk factors associated with this phenomenon, because the odds of the second-litter syndrome increase with litter size at parity 1 [6]. The decrease of litter size or farrowing rate in the second parity sows is often related to an excessive weight loss during first lactation [7, 8]. Other factors such as herd size, season of farrowing, and weaning to service interval have been reported as risk factors for the second-litter syndrome [6]. 

The pig industry is a very important activity in southern Mexico. To our knowledge, there are no reports of the incidence of and factors related to the second-litter syndrome in sows under tropical conditions. Therefore, the objectives of this study were to estimate the incidence of sows with the occurrence of the second-litter syndrome and to determine the effect of some factors, in three farms in the south eastern of Mexico.

## 2. Material and Methods

Data from three commercial farms of the state of Yucatan, Mexico, were used. Yucatan is localized at latitude 19°30′ and 21°35′N and longitude 90°24′W. The climate of the region is subhumid tropical, with an average temperature of 26.6°C, an average rainfall of 1,100 mm, and a relative humidity of 78% [9]. Pig production is the second more important livestock activity in Yucatan, being carried out under intensive conditions. There are approximately 105 farms with capacity for 28 to 3,500 sows. 

Farms 1, 2, and 4 were full cycle farms with 3,900, 1,200 and 550 sows, respectively. Farm 3 was a two-site-type farm (breeding and production) with 320 sows. The four farms produced their own replacements and practiced the quarantine of the gilts. Sows were fed commercial feed according to the productive stages. Young sows were given approximately 2.6 kg/day of a feed with 3,000 kcal EM/kg, 16% crude protein and 0.8% lysine, whereas the sows received 3.2 kg/day of feed. In all farms, breeding was carried out mainly by artificial insemination. After estrous detection using a boar, sows were inseminated three times every 12 hours.

Data from 2003 to 2011 recorded in the PIGCHAMP program were used. The information obtained was farm identification, sow identification, date of farrowing, number of piglets born alive (at first and second parity), and date of weaning. The weaning to conception interval was calculated as the length of the farrowing interval minus the average (115 days) gestation length [10]. 

The response variable was the second-litter syndrome, and it was defined as the sow with the same or lower numbers of pigs born alive in parity 2 as compared to parity 1 [2]. Sows were categorized into two groups: 1 if sows had similar or lower number of pigs at the second parity than that at the first parity and 0 for sows that increased litter size at the second parity. The data from 8,592 farrowing records for 4,296 sows were analyzed using binary logistic regression procedures. The risk factors evaluated were farm (1, 2, and 3), year of farrowing (2003–2011), season of farrowing (Dry, rainy and windy), number of pigs born alive (≤8, 9-10, 11-12, and ≥13 piglets), lactation length (≤17, 18–24, and ≥25 days), and weaning to conception intervals (≤3, 4–11, and ≥12 days). Seasons were categorized based on temperature and rainfall in the region, during the year. All statistical analyses were carried out with the SPSS program [11]. To declare significant effects *P* < 0.05 values were used.

## 3. Results

The overall frequency of sows with the second-litter syndrome was 55.8%, and the frequencies for farm 1, 2, and 3 were 60.4%, 52.2%, and 52.3%, respectively. The litter size means for farms 1, 2, and 3 and for parities 1 and 2 were 12.5 and 9.58, 10.9 and 8.09, and 10.4 and 8.08 pigs, respectively.

There were significant effects of farm, year of farrowing, season of farrowing, number of pigs born alive, and weaning to conception interval on the second-litter syndrome (Table 1). However, there was no effect of lactation length (or weaning age) of the sow (*P* > 0.05). The odds of the occurrence of the second-litter syndrome were 1.56 and 2.01 times higher for farms 2 and 3 as compared with farm 1. There was not any particular trend in the second-litter syndrome with years. The odds of the second-litter syndrome were 1.20 and 1.24 times higher for the sows farrowing during the dry and rainy seasons versus those farrowing in the windy season. Sows with large litters (>12 pigs) had higher odds (33.2) showing the second-litter syndrome than sows with small litters (<9 pigs). Sows with shorter weaning to conception intervals (<4 and 4–11 days) had higher odds showing a decrease in litter size at the second parity, as compared with sows with longer weaning to conception intervals.

## 4. Discussion

The overall frequency of sows showing the second-litter syndrome (55.8%) in the three farms, here studied, is higher than the 49.5% reported by Saito et al. [12] in 106 farms in Japan and the 54% reported by Morgan Morrow et al. [2] in 122 farms in the United States. However, the frequency of sows showing a decrease in litter size at the second parity, in this study, is within the range of reports in different countries, where 40 to 60% of the second-parity sows are affected [3, 12–14]. This may partially attributed to the use of highly prolific sows as a result of genetic improvement of litter size. In this study, 74.5% of the sows produced litter with 9 or more pigs. It is known that larger litter at birth had higher stillbirth losses as compared to small litters [15]. Therefore, farm productivity could be improved through the better management and care of prolific first parity sows.

Farm differences in the incidence of the second-litter syndrome were expected because of animal management differences, and biosecurity measures. Year of first farrowing effect, provides information on trends or fluctuations in the traits of interest with time; associated among others to management practices and owner policies. Year differences in the frequency of sows showing the second-litter syndrome have been notified by Boulot et al. [6] in France.

The higher odds of the second-litter syndrome for sows farrowing in the dry and rainy seasons may be attributed to the effect of the high temperature and/or high humidity in those seasons as compared with the cooler temperatures in the windy season (Table 1). The dry season in the region of study is characterized by high temperatures (up to 43°C) and relatively low humidity (up 65%), whereas in the rainy season the temperature and humidity reach values up to 37 and 100%, respectively. Whittemore [16] found that litters farrowed between October and March were 0.3 pigs larger (*P* < 0.05) than those farrowed between April and September in the Yorkshire breed. Season effect on the second-litter syndrome has also been reported by Boulot et al. [6] in France. 

The higher probability of a reduction in the size of the second litter, as the size of the first litter increases, agrees with the previous results [2, 6]. Those authors reported that large litter-size at the first parity is the main factor associated with the lower number of pigs born alive at the second parity. The use in farms of highly prolific sows with short weaning to estrus intervals may cause low ovulation rate and high embryonic mortality related to higher body weight losses during lactation [17]. Therefore, given that the odds (33.2) showing a reduction in the size of the second litter was greater for the first parity sows with large litters (>12 pigs), more attention should be given to them, with respect to feeding and body condition during lactation. Primiparous sows require higher demand of nutrients since they have not yet reached adult size and weight, and without a satisfactory feed intake during lactation they lost body weight which may cause high embryo mortality [18] and smaller litter size.

Morgan Morrow et al. [2] reported that delaying the breeding of the first parity sows after weaning was associated with the increased number of pigs born alive in parity 2. Koketsu et al. [19] and Rathje and Himmelberg [13] showed that for each additional day in lactation the subsequent litter size increases in 0.1 piglets. In this study, a decrease in the second litter was more likely for sows with prolonged weaning to conception interval, which agrees with the findings of Morgan Morrow et al. [2]. An approach to allow the first litter sow to recover from the previous lactation is to inseminate the sow at the second heat after weaning instead of the first one (skip a heat). Skipping the first heat can improve pregnancy rates by 15% and subsequently the litter sizes by 1.3 to 2.5 piglets [20, 21]. However, skipping a heat will increase the nonproductive days of the sows. 

From the results of this study, it can be concluded that a high proportion of sows showed the second-litter syndrome (55.8%). There were farm differences in the occurrence of the second-litter syndrome. Sows with large litters at the first parity, those farrowing in the dry or rainy seasons and those with shorter weaning to conception intervals, had higher odds showing the second-litter syndrome.

## Figures and Tables

**Table 1:** Factors associated with the second-litter syndrome in three pig farms in Yucatan, Mexico, using a binomial logistic model.

Factor	*N*	Estimate	Standard error	Odds ratio	95% Confidence limits
Farm					
1	1893	0		1	
2	843	0.442	0.105	1.55	1.26, 1.91
3	1560	0.697	0.092	2.01	1.68, 2.41

Year of farrowing					
2003	363	0		1	
2004	307	0.166	0.182	1.18	0.83, 1.69
2005	600	0.504	0.158	1.66	1.21, 2.26
2006	414	0.232	0.168	1.26	0.91, 1.75
2007	418	0.129	0.169	1.14	0.82, 1.59
2008	405	0.114	0.171	1.12	0.80, 1.57
2009	623	0.040	0.158	1.04	0.76, 1.42
2010	577	0.258	0.158	1.29	0.95, 1.76
2011	589	0.224	0.156	1.25	0.92, 1.70

Season of farrowing					
Dry	1360	0.180	0.087	1.20	1.01, 1.42
Rainy	1381	0.212	0.090	1.24	1.04, 1.47
Windy	1555	0		1	

Number of pigs born alive					
<9	1097	0		1	
9-10	1179	1.29	0.096	3.63	3.01, 4.38
11-12	1086	2.25	0.105	9.51	7.75, 11.7
>12	934	3.50	0.134	33.24	25.6, 43.2

Lactation length (days)					
<18	176	0.323	0.205	1.38	0.92, 2.06
18–24	3631	0.203	0.115	1.23	0.98, 1.54
>24	489	0		1	

Weaning to conception interval (days)					
<4	1505	0.577	0.091	1.78	1.49, 2.13
4–11	1344	1.01	0.092	2.74	2.29, 3.28
>11	1447	0		1	

## References

[1] Lawlor PG, Lynch PB (2007). A review of factors influencing litter size in Irish sows. *Irish Veterinary Journal*.

[2] Morgan Morrow WE, Leman AD, Williamson NB, Morrison RB, Ashley Robinson R (1992). An epidemiological investigation of reduced second-litter size in swine. *Preventive Veterinary Medicine*.

[3] Kemp B, Soede NM Reproductive problems in primiparous sows.

[4] Lucia T, Dial GD, Marsh WE (2000). Lifetime reproductive performance in female pigs having distinct reasons for removal. *Livestock Production Science*.

[5] Segura-Correa JC, Ek-Mex E, Alzina-López A, Segura-Correa VM (2011). Frequency of removal reasons of sows in Southeastern Mexico. *Tropical Animal Health and Production*.

[6] Boulot S, Despres Y, Badouard B, Sallé E Le “syndrome de 2eme portée” dans le élevages français: prévalence de différents profils et facteurs de risque. http://www.journees-recherche-porcine.com/gb/index.htm.

[7] Thaker MYC, Bilkei G (2005). Lactation weight loss influences subsequent reproductive performance of sows. *Animal Reproduction Science*.

[8] Schenkel AC, Bernardi ML, Bortolozzo FP, Wentz I (2010). Body reserve mobilization during lactation in first parity sows and its effect on second litter size. *Livestock Science*.

[9] Instituto Nacional de Estadística y Geografía (INEGI) Pespectivas estadística Yucatán. http://www.inegi.org.mx/prod_serv/contenidos/espanol/bvinegi/productos/integracion/estd_perspect/yuc/Pers-yuc.pdf.

[10] Ramírez-Gómez R, Segura Correa YJC (1991). Factores que afectan el período de gestación e intervalo entre partos de una piara comercial al noreste de México. *Livestock Research For Rural Development*.

[11] SPSS (2006). *SPSS for Windows Version 15*.

[12] Saito H, Sasaki Y, Hoshino Y, Koketsu Y (2010). The occurrence of decreased numbers of pigs born alive in parity 2 sows does not negatively affect herd productivity in Japan. *Livestock Science*.

[13] Rathje T, Himmelberg L Emerging technologies in reproduction: how the danes have reached 30 pigs/sow/year. http://www.aasv.org/library/swineinfo/Content/AASV/2004/395.pdf.

[14] Vargas AJ, Bernardi ML, Wentz I, Neto GB, Bortolozzo FP (2006). Time of ovulation and reproductive performance over three parities after treatment of primiparous sows with PG600. *Theriogenology*.

[15] Lucia T, Corrêa MN, Deschamps JC (2002). Risk factors for stillbirths in two swine farms in the south of Brazil. *Preventive Veterinary Medicine*.

[16] Whittemore CT (1996). Nutrition reproduction interactions in primiparous sows. *Livestock Production Science*.

[17] Southwood OI, Kennedy BW (1991). Genetic and environmental trends for litter size in swine. *Journal of Animal Science*.

[18] Hoving LL, Soede NM, Feitsma H, Kemp B (2012). Lactation weight loss in primiparous sows: consequences for embryo survival and progesterone and relations with metabolic profiles. *Reproduction in Domestic Animals*.

[19] Koketsu Y, Dial GD, Pettigrew JE, Marsh WE, King VL (1996). Influence of imposed feed intake patterns during lactation on reproductive performance and on circulating levels of glucose, insulin, and luteinizing hormone in primiparous sows. *Journal of Animal Science*.

[20] Clowes EJ, Aherne FX, Foxcroft GR (1994). Effect of delayed breeding on the endocrinology and fecundity of sows. *Journal of Animal Science*.

[21] Vesseur PC, Kemp B, den Hartog LA (1994). The effect of the weaning to estrus interval on litter size, live born piglets and farrowing rate in sows. *Journal of Animal Physiology and Animal Nutrition*.

